# Supplementary material for: The Influence of Baseline Clinical Status and Surgical Strategy on Early Good to Excellent Result in Spinal Lumbar Arthrodesis: A Machine Learning Approach
Source: J Pers Med. 2021 Dec 16;11(12):1377. doi: 10.3390/jpm11121377 (PMC8705358; doi:10.3390/jpm11121377)
Supplement: Supplementary file 1 [file jpm-11-01377-s001.zip › jpm-1507135-supplementary.pdf]

Table S1. Table 2x2 for Good Clinical Outcome

Gender

	Age	Outcome–	Outcome+
F	All	255 (19.30%)	568 (43.00%)
	20 - 64	165 (18.31%)	380 (42.18%)
	65 - 74	55 (19.10%)	130 (45.14%)
	>= 75	35 (28.46%)	53 (43.09%)
M	All	139 (10.52%)	359 (27.18%)
	20 - 64	101 (11.21%)	255 (28.30%)
	65 - 74	31 (10.76%)	72 (25.00%)
	>= 75	5 (4.07%)	30 (24.39%)

BMI (1)

	Age	Outcome–	Outcome+
< 25	All	182 (14.13%)	456 (35.40%)
	20 - 64	137 (15.53%)	342 (38.78%)
	65 - 74	26 (9.39%)	72 (25.99%)
	>= 75	17 (14.17%)	37 (30.83%)
>= 25	All	199 (15.45%)	451 (35.02%)
	20 - 64	124 (14.06%)	279 (31.63%)
	65 - 74	53 (19.13%)	126 (45.49%)
	>= 75	22 (18.33%)	44 (36.67%)

BMI (2)

	Age	Outcome–	Outcome+
< 30	All	343 (26.63%)	780 (60.56%)
	20 - 64	238 (26.98%)	550 (62.36%)
	65 - 74	65 (23.47%)	157 (56.68%)
	>= 75	38 (31.67%)	68 (56.67%)
>= 30	All	38 (2.95%)	127 (9.86%)
	20 - 64	23 (2.61%)	71 (8.05%)
	65 - 74	14 (5.05%)	41 (14.80%)
	>= 75	1 (0.83%)	13 (10.83%)

Revisions

	Age	Outcome–	Outcome+
No	All	116 (14.78%)	397 (50.57%)
	20 - 64	80 (14.95%)	257 (48.04%)
	65 - 74	23 (13.45%)	98 (57.31%)
	>= 75	13 (17.57%)	38 (51.35%)
Sì	All	108 (13.76%)	164 (20.89%)
	20 - 64	75 (14.02%)	123 (22.99%)
	65 - 74	23 (13.45%)	27 (15.79%)
	>= 75	9 (12.16%)	14 (18.92%)

Charlson Index (1)

	Age	Outcome–	Outcome+
< 5	All	118 (15.07%)	298 (38.06%)
	20 - 64	102 (19.14%)	243 (45.59%)
	65 - 74	10 (5.85%)	38 (22.22%)
	>= 75	5 (6.76%)	13 (15.57%)
>= 5	Tutti	105 (13.41%)	262 (33.46%)
	20 - 64	52 (9.76%)	136 (25.52%)
	65 - 74	36 (21.05%)	87 (50.88%)
	>= 75	17 (22.97%)	39 (52.70%)

Charlson Index (2)

	Age	Outcome–	Outcome+
< 2	All	0 (0.00%)	0 (0.00%)
	20 - 64	0 (0.00%)	0 (0.00%)
	65 - 74	0 (0.00%)	0 (0.00%)
	>= 75	0 (0.00%)	0 (0.00%)
>= 2	All	223 (28.48%)	560 (71.52%)
	20 - 64	154 (28.89%)	379 (71.11%)
	65 - 74	46 (26.9%)	125 (73.1%)
	>= 75	22 (29.73%)	52 (70.27%)

Livels (1)

	Age	Outcome–	Outcome+
< 3	All	142 (18.91%)	417 (55.53%)
	20 - 64	111 (21.81%)	291 (57.17%)
	65 - 74	21 (12.96%)	79 (48.77%)
	>= 75	10 (13.33%)	43 (57.33%)
>= 3	All	77 (10.25%)	115 (15.31%)
	20 - 64	38 (7.47%)	69 (13.56%)
	65 - 74	25 (15.43%)	37 (22.84%)
	>= 75	13 (17.33%)	9 (12.00%)

Livels (2)

	Age	Outcome–	Outcome+
< 2	All	51 (6.79%)	180 (23.97%)
	20 - 64	44 (8.64%)	137 (26.92%)
	65 - 74	4 (2.47%)	26 (16.05%)
	>= 75	3 (4.00%)	16 (21.33%)
>= 2	All	168 (22.37%)	352 (46.87%)
	20 - 64	105 (20.63%)	223 (43.81%)
	65 - 74	42 (25.93%)	90 (55.56%)
	>= 75	20 (26.67%)	36 (48.00%)

L5 – S1

	Age	Outcome–	Outcome+
Other	All	169 (22.44%)	380 (50.46%)
	20 - 64	102 (20.08%)	231 (45.47%)
	65 - 74	43 (26.06%)	103 (62.42%)
	>= 75	23 (30.67%)	46 (61.33%)
L5 – S1	All	50 (6.64%)	154 (20.45%)
	20 - 64	46 (9.06%)	129 (25.39%)
	65 - 74	4 (2.42%)	15 (9.09%)
	>= 75	0 (0.00%)	6 (8.00%)

L4 – L5

	Age	Outcome–	Outcome+
Other	All	179 (23.77%)	378 (50.20%)
	20 - 64	118 (23.23%)	255 (50.20%)
	65 - 74	42 (25.45%)	86 (52.12%)
	>= 75	18 (24.00%)	33 (44.00%)
L4 – L5	All	40 (5.31%)	156 (20.72%)
	20 - 64	30 (5.91%)	105 (20.67%)
	65 - 74	5 (3.03%)	32 (19.39%)
	>= 75	5 (6.67%)	19 (25.33%)

L3 – L4

	Age	Outcome–	Outcome+
Other	All	206 (27.36%)	497 (66.00%)
	20 - 64	142 (27.95%)	343 (67.52%)
	65 - 74	43 (26.06%)	105 (63.64%)
	>= 75	20 (26.67%)	45 (60.00%)
L3 – L4	All	13 (1.73%)	37 (4.91%)

L2 – L3

	Age	Outcome–	Outcome+
Other	All	217 (28.82%)	533 (70.78%)
	20 - 64	147 (28.94%)	360 (70.87%)
	65 - 74	46 (27.88%)	117 (70.91%)
	>= 75	23 (30.67%)	52 (69.33%)
L2 – L3	All	2 (0.27%)	1 (0.13%)

	20 - 64	6 (1.18%)	17 (3.35%)
	65 - 74	4 (2.42%)	13 (7.88%)
	>= 75	3 (4.00%)	7 (9.33%)

	20 – 64	1 (0.20%)	0 (0.00%)
	65 – 74	1 (0.61%)	1 (0.61%)
	>= 75	0 (0.00%)	0 (0.00%)

L1 – L2	Age	Outcome–	Outcome+
Other	All	218 (28.95%)	532 (70.65%)
	20 - 64	147 (28.94%)	359 (70.67%)
	65 - 74	47 (28.48%)	117 (70.91%)
	>= 75	23 (30.67%)	52 (69.33%)
L1 – L2	All	1 (0.13%)	2 (0.27%)
	20 - 64	1 (0.20%)	1 (0.20%)
	65 - 74	0 (0.00%)	1 (0.61%)
	>= 75	0 (0.00%)	0 (0.00%)

Number of Prevoius Surgeries	Age	Outcome–	Outcome+
< 3	All	203 (25.86%)	540 (68.79%)
	20 - 64	138 (25.79%)	362 (67.66%)
	65 - 74	42 (24.56%)	123 (71.93%)
	>= 75	22 (29.73%)	51 (68.92%)
>= 3	All	21 (2.68%)	21 (2.68%)
	20 - 64	17 (3.18%)	18 (3.36%)
	65 - 74	4 (2.34%)	2 (1.17%)
	>= 75	0 (0.00%)	1 (1.35%)

Gender: Female (F) vs. Male (M); BMI (1): Body Mass Index <25 vs. >= 25; BMI (2): Body Mass Index <30 vs. >= 30; Revision: Surgical revision; Charlson Index (1): < 5 diseases vs. >= 5 diseases; ; Charlson Index (2): < 2 diseases vs. >= 2 diseases; Levels (1): <3 levels of surgery vs. >= 3 levels of surgery; L5-S1: operated vs. no operated; L4-L5: operated vs. no operated; L3-L4: operated vs. no operated; L2-L3: operated vs. no operated; L1-L2: operated vs. no operated; Number of Prevoius Surgeries: <3 previous surgeries vs. >= 3 previous surgeries.

Table S2. Odds ratio for Good Clinical Outcome

	Outcome	
Factor	+	-
*	A	B
**	C	D

$$OR = \frac{A/B}{C/D} = \frac{AD}{BC}$$

	All	20 – 64	65 – 74	>= 75
Gender	0.86 (0.67, 1.10)	0.91 (0.68, 1.22)	1.02 (0.60, 1.72)	0.25 (0.09, 0.71)
BMI (1)	1.11 (0.87, 1.40)	1.11 (0.83, 1.48)	1.16 (0.67, 2.02)	1.09 (0.50, 2.35)
BMI (2)	0.68 (0.46, 1.00)	0.75 (0.46, 1.23)	0.82 (0.42, 1.62)	0.14 (0.02, 1.09)
Revisions	2.25 (1.64, 3.10)	1.96 (1.34, 2.87)	3.63 (1.77, 7.44)	1.88 (0.66, 5.36)
Charlson (1)	1.01 (0.74, 1.38)	0.91 (0.61, 1.35)	1.57 (0.71, 3.49)	1.13 (0.35, 3.68)
Charlson (2)	-	-	-	-
Livels (1)	1.97 (1.39, 2.78)	1.44 (0.92, 2.27)	2.54 (1.26, 5.12)	6.21 (2.08, 18.54)
Livels (2)	1.68 (1.17, 2.42)	1.47 (0.97, 2.21)	3.03 (1.00, 9.25)	2.96 (0.77, 11.42)
L5-S1	0.73 (0.51, 1.05)	0.81 (0.54, 1.22)	0.64 (0.20, 2.04)	-
L4-L5	0.54 (0.37, 0.80)	0.62 (0.39, 0.98)	0.32 (0.12, 0.88)	0.48 (0.15, 1.51)
L3-L4	0.85 (0.44, 1.63)	0.85 (0.33, 2.21)	0.75 (0.23, 2.43)	-
L2-L3	-	-	-	-
L1-L2	-	-	-	-
Number of Previous Surgeries	2.66 (1.42, 4.97)	2.48 (1.24, 4.95)	-	-

OR (CI); Gender: Female (F) vs. Male (M); BMI (1): Body Mass Index <25 vs. >= 25; BMI (2): Body Mass Index <30 vs. >= 30; Revision: Surgical revision; Charlson Index (1): < 5 diseases vs. >= 5 diseases; ; Charlson Index (2): < 2 diseases vs. >= 2 diseases; Levels (1): <3 levels of surgery vs. >= 3 levels of surgery; L5-S1: operated vs. no operated; L4-L5: operated vs. no operated; L3-L4: operated vs. no operated; L2-L3: operated vs. no operated; L1-L2: operated vs. no operated; Number of Prevoius Surgeries: <3 previous surgeries vs. >= 3 previous surgeries.

Table S3. Chi-square test for Good Clinical Outcome

Overall

	Gender	BMI (1)	BMI (2)	Revisions	Charlson Index (1)	Charlson Index (2)	Livels (1)
p-value	0.2623157	0.4471738	0.05969474	6.916487e-07	1	-	0.0001600744
conclusion	No diff	No diff	No diff	Diff	No diff	-	Diff

	Livels (2)	L5-S1	L4-L5	L3-L4	L2-L3	L1-L2	Number of Previous Surgeries
p-value	0.005788207	0.1108504	0.002543689	0.7370483	-	-	0.002782985
conclusion	Diff	No diff	Diff	No diff	-	-	Diff

Age: 20 - 64

	Gender	BMI (1)	BMI (2)	Revisions	Charlson Index (1)	Charlson Index (2)	Livels (1)
p-value	0.5905967	0.529602	0.302134	0.0007186022	0.7160197	-	0.1396932
conclusion	No diff	No diff	No diff	Diff	No diff	-	No diff

	Livels (2)	L5-S1	L4-L5	L3-L4	L2-L3	L1-L2	Number of Previous Surgeries
p-value	0.08425418	0.356826	0.05093399	0.9248703	-	-	0.0142337
conclusion	No diff	No diff	No diff	No diff	-	-	Diff

Age: 65 - 74

	Gender	BMI (1)	BMI (2)	Revisions	Charlson Index (1)	Charlson Index (2)	Livels (1)
p-value	1	0.6866514	0.6923901	0.0006014239	0.3545524	-	0.01344484
conclusion	No diff	No diff	No diff	Diff	No diff	-	Diff

	Livels (2)	L5-S1	L4-L5	L3-L4	L2-L3	L1-L2	Number of Previous Surgeries
p-value	0.07146174	0.6221021	0.03715548	0.8459507	-	-	-
conclusion	No diff	No diff	Diff	No diff	-	-	-

Age: >= 75

	Gender	BMI (1)	BMI (2)	Revisions	Charlson Index (1)	Charlson Index (2)	Livels (1)
p-value	0.01209824	0.9843717	0.06406321	0.3610345	1	-	0.001553829
conclusion	Diff	No diff	No diff	No diff	No diff	-	Diff

	Livels (2)	L5-S1	L4-L5	L3-L4	L2-L3	L1-L2	Number of Previous Surgeries
p-value	0.1803609	-	0.3180364	-	-	-	-
conclusion	No diff	-	No diff	-	-	-	-

$H0: \pi_1 = \pi_2$ There is no difference between the proportions (No diff); $H1: \pi_1 \neq \pi_2$ Significant difference in proportions (Diff); Note: significance of 5%. Gender: Female (F) vs. Male (M); BMI (1): Body Mass Index <25 vs. >= 25; BMI (2): Body Mass Index <30 vs. >= 30; Revision: Surgical revision; Charlson Index (1): < 5 diseases vs. >= 5 diseases; ; Charlson Index (2): < 2 diseases vs. >= 2 diseases; Levels (1): <3 levels of surgery vs. >= 3 levels of surgery; L5-S1: operated vs. no operated; L4-L5: operated vs. no operated; L3-L4: operated vs. no operated; L2-L3: operated vs. no operated; L1-L2: operated vs. no operated; Number of Prevoius Surgeries: <3 previous surgeries vs. >= 3 previous surgeries.

Table S4. Summary table of the chi-square test for Good Clinical Outcome

	Gender	BMI (1)	BMI (2)	Revisions	Charlson Index (1)	Charlson Index (2)	Livels (1)	Livels (2)	L5-S1	L4-L5	L3-L4	L2-L3	L1-L2	Number of Previous Surgeries
All	No	No	No	Diff	No	-	Diff	Diff	No	Diff	No	-	-	Diff
20 – 64	No	No	No	Diff	No	-	No	No	No	No	No	-	-	Diff
65 – 74	No	No	No	Diff	No	-	Diff	No	No	Diff	No	-	-	-
>= 75	Diff	No	No	No	No	-	Dif	No	-	No	-	-	-	-

No: no significant difference / Diff: significant difference / -: presence of zero or almost zero frequencies. Gender: Female (F) vs. Male (M); BMI (1): Body Mass Index <25 vs. >= 25; BMI (2): Body Mass Index <30 vs. >= 30; Revision: Surgical revision; Charlson Index (1): < 5 diseases vs. >= 5 diseases; ; Charlson Index (2): < 2 diseases vs. >= 2 diseases; Levels (1): <3 levels of surgery vs. >= 3 levels of surgery; L5-S1: operated vs. no operated; L4-L5: operated vs. no operated; L3-L4: operated vs. no operated; L2-L3: operated vs. no operated; L1-L2: operated vs. no operated; Number of Prevoius Surgeries: <3 previous surgeries vs. >= 3 previous surgeries.

Table S5. Table 2x2 for Excellent Clinical Outcome

Gender	Age	Outcome–	Outcome+
F	All	433 (34.98%)	338 (27.30%)
	20 - 64	279 (33.37%)	223 (26.67%)
	65 - 74	99 (36.13%)	79 (28.83%)
	>= 75	55 (46.22%)	31 (26.05%)
M	All	246 (19.87%)	221 (17.85%)
	20 - 64	178 (21.29%)	156 (18.66%)
	65 - 74	46 (16.79%)	50 (18.25%)
	>= 75	18 (15.13%)	15 (12.61%)

BMI (1)	Age	Outcome–	Outcome+
< 25	All	327 (27.11%)	264 (21.89%)
	20 - 64	247 (30.20%)	195 (23.84%)
	65 - 74	48 (18.25%)	44 (16.73%)
	>= 75	28 (24.14%)	22 (18.97%)
>= 25	All	333 (27.61%)	282 (23.38%)
	20 - 64	202 (24.69%)	174 (21.27%)
	65 - 74	88 (33.46%)	83 (31.56%)
	>= 75	43 (37.07%)	23 (19.83%)

BMI (2)	Age	Outcome–	Outcome+
< 30	All	583 (48.34%)	468 (38.81%)
	20 - 64	407 (49.76%)	327 (39.98%)
	65 - 74	106 (40.30%)	102 (38.78%)
	>= 75	66 (56.90%)	36 (31.03%)
>= 30	All	77 (6.38%)	78 (6.47%)
	20 - 64	42 (5.13%)	42 (5.13%)
	65 - 74	30 (11.41%)	25 (9.51%)
	>= 75	5 (4.31%)	9 (7.76%)

Revisions	Age	Outcome–	Outcome+
No	All	229 (31.16%)	254 (34.56%)
	20 - 64	156 (31.45%)	159 (32.06%)
	65 - 74	44 (27.16%)	71 (43.83%)
	>= 75	28 (38.89%)	21 (29.17%)
Yes	All	163 (22.18%)	89 (12.11%)
	20 - 64	110 (22.18%)	71 (14.31%)
	65 - 74	35 (21.60%)	12 (7.41%)
	>= 75	17 (23.61%)	6 (8.33%)

Charlson (1)	Age	Outcome–	Outcome+
< 5	All	208 (28.38%)	177 (24.15%)
	20 - 64	176 (35.63%)	141 (28.54%)
	65 - 74	20 (12.35%)	25 (15.43%)
	>= 75	10 (13.89%)	8 (11.11%)
>= 5	All	183 (24.97%)	165 (22.51%)
	20 - 64	89 (18.02%)	88 (17.81%)
	65 - 74	59 (36.42%)	58 (35.80%)
	>= 75	35 (48.61%)	19 (26.39%)

Charlson (2)	Age	Outcome–	Outcome+
< 2	All	0 (0.00%)	0 (0.00%)
	20 - 64	0 (0.00%)	0 (0.00%)
	65 - 74	0 (0.00%)	0 (0.00%)
	>= 75	0 (0.00%)	0 (0.00%)
>= 2	All	391 (53.34%)	342 (46.66%)
	20 - 64	265 (53.64%)	229 (46.36%)
	65 - 74	79 (48.77%)	83 (51.23%)
	>= 75	45 (62.5%)	27 (37.5%)

Livelli (1)	Age	Outcome–	Outcome+
< 3	All	254 (36.03%)	271 (38.44%)
	20 - 64	189 (39.87%)	186 (39.24%)
	65 - 74	36 (23.53%)	58 (37.91%)
	>= 75	28 (38.36%)	24 (32.88%)
>= 3	All	126 (17.87%)	54 (7.66%)
	20 - 64	67 (14.14%)	32 (6.75%)
	65 - 74	40 (26.14%)	19 (12.42%)
	>= 75	18 (24.66%)	3 (4.11%)

Livelli (2)	Age	Outcome–	Outcome+
< 2	All	97 (13.76%)	121 (17.16%)
	20 - 64	80 (16.88%)	90 (18.99%)
	65 - 74	7 (4.58%)	21 (13.73%)
	>= 75	10 (13.70%)	9 (12.33%)
>= 2	All	283 (40.14%)	204 (28.94%)
	20 - 64	176 (37.13%)	128 (27.00%)
	65 - 74	69 (45.10%)	56 (36.60%)
	>= 75	36 (49.32%)	18 (24.66%)

L5 – S1	Age	Outcome–	Outcome+
Other	All	284 (40.23%)	227 (32.15%)
	20 - 64	170 (36.02%)	135 (28.60%)
	65 - 74	69 (44.23%)	69 (44.23%)
	>= 75	44 (60.27%)	23 (31.51%)
L5 – S1	All	95 (13.46%)	100 (14.16%)
	20 - 64	84 (17.80%)	83 (17.58%)
	65 - 74	8 (5.13%)	10 (6.41%)
	>= 75	2 (2.74%)	4 (5.48%)

L4 – L5	Age	Outcome–	Outcome+
Other	All	304 (43.06%)	220 (31.16%)
	20 - 64	202 (42.80%)	146 (30.93%)
	65 - 74	69 (44.23%)	53 (33.97%)
	>= 75	31 (42.47%)	18 (24.66%)
L4 – L5	All	75 (10.62%)	107 (15.16%)
	20 - 64	52 (11.02%)	72 (15.25%)
	65 - 74	8 (5.13%)	26 (16.67%)
	>= 75	15 (20.55%)	9 (12.33%)

L3 – L4	Age	Outcome–	Outcome+
Other	All	359 (50.85%)	301 (42.63%)
	20 - 64	245 (51.91%)	207 (43.86%)
	65 - 74	71 (45.51%)	69 (44.23%)
	>= 75	41 (56.16%)	22 (30.14%)
L3 – L4	All	20 (2.83%)	26 (3.68%)

L2 – L3	Age	Outcome–	Outcome+
Other	All	377 (53.40%)	326 (46.18%)
	20 - 64	253 (53.60%)	218 (46.19%)
	65 - 74	76 (48.72%)	78 (50.00%)
	>= 75	46 (63.01%)	27 (36.99%)
L2 – L3	All	2 (0.28%)	1 (0.14%)

	20 - 64	9 (1.91%)	11 (2.33%)
	65 - 74	6 (3.85%)	10 (6.41%)
	>= 75	5 (6.85%)	5 (6.85%)

	20 – 64	1 (0.21%)	0 (0.00%)
	65 – 74	1 (0.64%)	1 (0.64%)
	>= 75	0 (0.00%)	0 (0.00%)

L1 – L2	Age	Outcome–	Outcome+
Other	All	376 (53.26%)	327 (46.32%)
	20 - 64	252 (53.39%)	218 (46.19%)
	65 - 74	76 (48.72%)	79 (50.64%)
	>= 75	46 (63.01%)	27 (36.99%)
L1 – L2	All	3 (0.42%)	0 (0.00%)
	20 - 64	2 (0.42%)	0 (0.00%)
	65 - 74	1 (0.64%)	0 (0.00%)
	>= 75	0 (0.00%)	0 (0.00%)

Number of Previous Surgeries	Age	Outcome–	Outcome+
< 3	All	360 (48.98%)	336 (45.71%)
	20 - 64	240 (48.39%)	223 (44.96%)
	65 - 74	74 (45.68%)	83 (51.23%)
	>= 75	44 (61.11%)	27 (37.50%)
>= 3	All	32 (4.35%)	7 (0.95%)
	20 - 64	26 (5.24%)	7 (1.41%)
	65 - 74	5 (3.09%)	0 (0.00%)
	>= 75	1 (1.39%)	0 (0.00%)

Gender: Female (F) vs. Male (M); BMI (1): Body Mass Index <25 vs. >= 25; BMI (2): Body Mass Index <30 vs. >= 30; Revision: Surgical revision; Charlson Index (1): < 5 diseases vs. >= 5 diseases; ; Charlson Index (2): < 2 diseases vs. >= 2 diseases; Levels (1): <3 levels of surgery vs. >= 3 levels of surgery; L5-S1: operated vs. no operated; L4-L5: operated vs. no operated; L3-L4: operated vs. no operated; L2-L3: operated vs. no operated; L1-L2: operated vs. no operated; Number of Prevoius Surgeries: <3 previous surgeries vs. >= 3 previous surgeries.

Table S6. Odds ratio for Excellent Clinical Outcome

	Outcome	
Factor	+	-
*	A	B
**	C	D

$$OR = \frac{A/B}{C/D} = \frac{AD}{BC}$$

	All	20 – 64	65 – 74	>= 75
Gender	0.87 (0.69, 1.09)	0.91 (0.69, 1.20)	0.73 (0.45, 1.21)	0.68 (0.30, 1.53)
BMI (1)	0.95 (0.76, 1.20)	0.92 (0.70, 1.21)	0.97 (0.59, 1.61)	1.47 (0.69, 3.12)
BMI (2)	0.79 (0.57, 1.11)	0.80 (0.51, 1.26)	1.15 (0.64, 2.10)	0.30 (0.09, 0.97)
Revisions	2.03 (1.48, 2.78)	1.58 (1.09, 2.29)	4.71 (2.21, 10.02)	2.12 (0.72, 6.32)
Charlson (1)	0.94 (0.71, 1.26)	0.81 (0.56, 1.17)	1.27 (0.64, 2.54)	1.47 (0.50, 4.36)
Charlson (2)	-	-	-	-
Livels (1)	2.49 (1.73, 3.57)	2.06 (1.29, 3.29)	3.39 (1.71, 6.74)	5.14 (1.35, 19.61)
Livels (2)	1.73 (1.25, 2.39)	1.55 (1.06, 2.26)	3.70 (1.47, 9.32)	1.80 (0.62, 5.21)
L5-S1	0.76 (0.55, 1.06)	0.80 (0.55, 1.17)	0.80 (0.30, 2.15)	0.26 (0.04, 1.54)
L4-L5	0.51 (0.36, 0.71)	0.52 (0.34, 0.79)	0.24 (0.10, 0.56)	0.97 (0.35, 2.66)
L3-L4	0.64 (0.35, 1.18)	0.69 (0.28, 1.70)	0.58 (0.20, 1.69)	0.54 (0.14, 2.06)
L2-L3	-	-	-	-
L1-L2	-	-	-	-
Number of Previous Surgeries	4.27 (1.86, 9.80)	3.45 (1.47, 8.11)	-	-

OR (CI)

OR: (CI); Gender: Female (F) vs. Male (M); BMI (1): Body Mass Index <25 vs. >= 25; BMI (2): Body Mass Index <30 vs. >= 30; Revision: Surgical revision; Charlson Index (1): < 5 diseases vs. >= 5 diseases; ; Charlson Index (2): < 2 diseases vs. >= 2 diseases; Levels (1): <3 levels of surgery vs. >= 3 levels of surgery; L5-S1: operated vs. no operated; L4-L5: operated vs. no operated; L3-L4: operated vs. no operated; L2-L3: operated vs. no operated; L1-L2: operated vs. no operated; Number of Prevoius Surgeries: <3 previous surgeries vs. >= 3 previous surgeries.

Table S7. Chi-square test for Excellent Clinical Outcome

Overall

	Gender	BMI (1)	BMI (2)	Revisions	Charlson Index (1)	Charlson Index (2)	Livels (1)
p-value	0.2563394	0.7226324	0.2053972	1.203415e-05	0.7519674	-	8.030184e-07
conclusion	No diff	No diff	No diff	Diff	No diff	-	Diff

	Livels (2)	L5-S1	L4-L5	L3-L4	L2-L3	L1-L2	Number of Previous Surgeries
p-value	0.001074997	0.1211771	0.000127610 1	0.1996269	-	-	0.0004166708
conclusion	Diff	No diff	Diff	No diff	-	-	Diff

Age: 20 - 64

	Gender	BMI (1)	BMI (2)	Revisions	Charlson Index (1)	Charlson Index (2)	Livels (1)
p-value	0.5626514	0.5837394	0.4036794	0.02006563	0.3051875	-	0.003131671
conclusion	No diff	No diff	No diff	Diff	No diff	-	Diff

	Livels (2)	L5-S1	L4-L5	L3-L4	L2-L3	L1-L2	Number of Previous Surgeries
p-value	0.02969463	0.2999077	0.002836307	0.5627606	-	-	0.004816911
conclusion	Diff	No diff	Diff	No diff	-	-	Diff

Age: 65 - 74

	Gender	BMI (1)	BMI (2)	Revisions	Charlson Index (1)	Charlson Index (2)	Livels (1)
p-value	0.2750078	1	0.7479786	6.04923e-05	0.6122256	-	0.0007091525
conclusion	No diff	No diff	No diff	Diff	No diff	-	Diff

	Livels (2)	L5-S1	L4-L5	L3-L4	L2-L3	L1-L2	Number of Previous Surgeries
p-value	0.007365971	0.8471254	0.001315736	0.4607421	-	-	-
conclusion	Diff	No diff	Diff	No diff	-	-	-

Age: >= 75

	Gender	BMI (1)	BMI (2)	Revisions	Charlson Index (1)	Charlson Index (2)	Livels (1)
p-value	0.4634132	0.4183245	0.07264382	0.2672363	0.67329	-	0.02229397
conclusion	No diff	No diff	No diff	No diff	No diff	-	Diff

	Livels (2)	L5-S1	L4-L5	L3-L4	L2-L3	L1-L2	Number of Previous Surgeries
p-value	0.4158479	-	1	-	-	-	-
conclusion	No diff	-	No diff	-	-	-	-

$H0: \pi_1 = \pi_2$ There is no difference between the proportions (No diff); $H1: \pi_1 \neq \pi_2$ Significant difference in proportions (Diff); Note: significance of 5%. Gender: Female (F) vs. Male (M); BMI (1): Body Mass Index <25 vs. >= 25; BMI (2): Body Mass Index <30 vs. >= 30; Revision: Surgical revision; Charlson Index (1): < 5 diseases vs. >= 5 diseases; ; Charlson Index (2): < 2 diseases vs. >= 2 diseases; Levels (1): <3 levels of surgery vs. >= 3 levels of surgery; L5-S1: operated vs. no operated; L4-L5: operated vs. no operated; L3-L4: operated vs. no operated; L2-L3: operated vs. no operated; L1-L2: operated vs. no operated; Number of Prevoius Surgeries: <3 previous surgeries vs. >= 3 previous surgeries.

Table S8. Summary table of the chi-square test for Excellent Clinical Outcome

	Gender	BMI (1)	BMI (2)	Revisions	Charlson Index (1)	Charlson Index (2)	Livels (1)	Livels (2)	L5-S1	L4-L5	L3-L4	L2-L3	L1-L2	Number of Previous Surgeries
Tutti	No	No	No	Diff	No	-	Diff	Diff	No	Diff	No	-	-	Dif
20 – 64	No	No	No	Diff	No	-	Diff	Diff	No	Diff	No	-	-	Dif
65 – 74	No	No	No	Diff	No	-	Diff	Diff	No	Diff	No	-	-	-
>= 75	No	No	No	No	No	-	Diff	No	-	No	-	-	-	-

No: no significant difference / Diff: significant difference / -: presence of zero or almost zero frequencies. Gender: Female (F) vs. Male (M); BMI (1): Body Mass Index <25 vs. >= 25; BMI (2): Body Mass Index <30 vs. >= 30; Revision: Surgical revision; Charlson Index (1): < 5 diseases vs. >= 5 diseases; ; Charlson Index (2): < 2 diseases vs. >= 2 diseases; Levels (1): <3 levels of surgery vs. >= 3 levels of surgery; L5-S1: operated vs. no operated; L4-L5: operated vs. no operated; L3-L4: operated vs. no operated; L2-L3: operated vs. no operated; L1-L2: operated vs. no operated; Number of Prevoius Surgeries: <3 previous surgeries vs. >= 3 previous surgeries.

Table S9. Paired t-test for Good Clinical Outcome

		ODI	COMI	SF36 P	SF36 M
All	p-value	4.982479e-248	9.081321e-313	1.096454e-152	5.228172e-38
	conclusion	Diff	Diff	Diff	Diff
20 – 64	p-value	7.038763e-170	4.931774e-210	2.07989e-99	3.502438e-22
	conclusion	Diff	Diff	Diff	Diff
65 – 74	p-value	1.10763e-59	1.279598e-72	2.761798e-41	9.231984e-17
	conclusion	Diff	Diff	Diff	Diff
>= 75	p-value	1.305395e-20	1.983991e-30	1.379067e-14	0.001794689
	conclusion	Diff	Diff	Diff	Diff

H0: There is no difference between the averages of the two paired samples (pre and post operation) (No diff); H1: Significant difference between the means of the two samples (pre and post operation) (Diff); Note: significance of 5%. ODI: Oswestry disability index; COMI: pre-operative core outcome measures index; SF36 P: pre-operative physical component score of the short form-36; SF36 M: pre-operative men-tal component score of the short form-36.

Table S10. Paired t-test for Excellent Clinical Outcome

		ODI	COMI	SF36 P	SF36 M
All	p-value	2.491239e-248	4.54066e-313	5.482269e-153	2.614086e-38
	conclusion	Pre > Post	Pre > Post	Pre < Post	Pre < Post
20 – 64	p-value	3.519381e-170	2.465887e-210	1.039945e-99	1.751219e-22
	conclusion	Pre > Post	Pre > Post	Pre < Post	Pre < Post
65 – 74	p-value	5.538152e-60	6.397991e-73	1.380899e-41	4.615992e-17
	conclusion	Pre > Post	Pre > Post	Pre < Post	Pre < Post
>= 75	p-value	6.526976e-21	9.919954e-31	6.895335e-15	0.0008973446
	conclusion	Pre > Post	Pre > Post	Pre < Post	Pre < Post

H0: There is no difference between the averages of the two paired samples (pre and post operation); H1: Pre operation> Post operation (ODI, COMI), Pre operation <Post operation (SF36 P / M); Note: significance of 5%. ODI: Oswestry disability index; COMI: pre-operative core outcome measures index; SF36 P: pre-operative physical component score of the short form-36; SF36 M: pre-operative men-tal component score of the short form-36.

Table S11. Wilcoxon test for Good and Excellent Clinical Outcome

		ODI	COMI	SF36 P	SF36 M
All	p-value	1.271228e-180	1.847914e-190	3.257621e-126	7.759346e-36
	conclusion	Diff	Diff	Diff	Diff
20 – 64	p-value	9.914093e-126	7.047989e-129	2.206261e-82	4.243412e-20
	conclusion	Diff	Diff	Diff	Diff
65 – 74	p-value	2.526231e-41	1.88675e-44	9.822779e-34	4.618583e-17
	conclusion	Diff	Diff	Diff	Diff
>= 75	p-value	2.728287e-16	1.958907e-19	4.893749e-13	0.002570207
	conclusion	Diff	Diff	Diff	Diff

		ODI	COMI	SF36 P	SF36 M

All	p-value	6.356139e-181	9.23957e-191	1.62881e-126	3.879673e-36
	conclusion	Pre > Post	Pre > Post	Pre < Post	Pre < Post
20 – 64	p-value	4.957046e-126	3.523994e-129	1.10313e-82	2.121706e-20
	conclusion	Pre > Post	Pre > Post	Pre < Post	Pre < Post
65 – 74	p-value	1.263115e-41	9.43375e-45	4.911389e-34	2.309292e-17
	conclusion	Pre > Post	Pre > Post	Pre < Post	Pre < Post
>= 75	p-value	1.364144e-16	9.794533e-20	2.446875e-13	0.001285104
	conclusion	Pre > Post	Pre > Post	Pre < Post	Pre < Post

Non-parametric equivalent of the paired t-test generally applied for data not satisfying normality. In this case it is applied to all data anyway.The differences in all pre- and post-operation scores are significant for all age scenarios. In ODI and COMI the pre-operation scores are significantly higher than the post-operation scores for all age scenarios, vice versa for SF36 PCS / MCS. ODI: Oswestry disability index; COMI: pre-operative core outcome measures index; SF36 P: pre-operative physical component score of the short form-36; SF36 M: pre-operative men-tal component score of the short form-36.

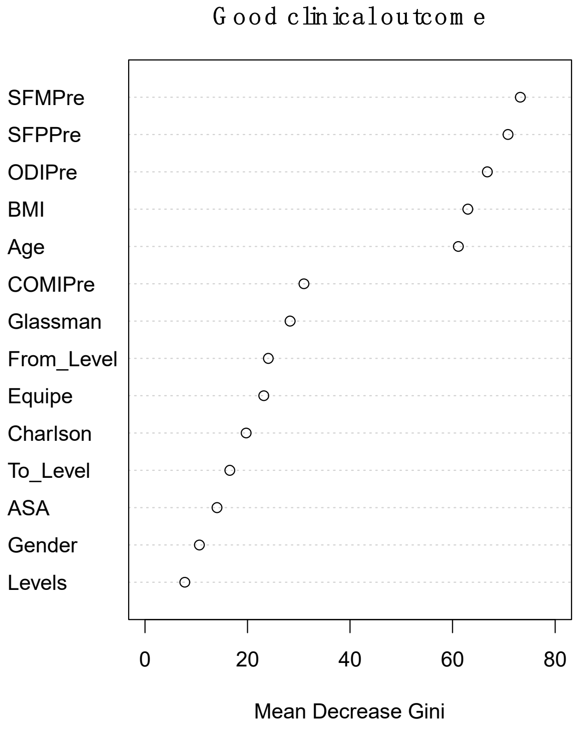

Figure S1. Mean Decrease Gini for Good Clinical Outcome. Glassman: Glassman classification data; Equipe: surgical team; BMI: body mass index; ODIPre: pre-operative; Oswestry disability index; COMIPre: pre-operative core outcome measures index; SFPPre: pre-operative physical component score of the short form-36; SFMPre: pre-operative men-tal component score of the short form-36; CCI: Charlson comorbidity index. From_level: Upper instrumented vertebra; To_level: lower level of instrumented vertebra. ASA: American Society of Anesthesiologists Score.

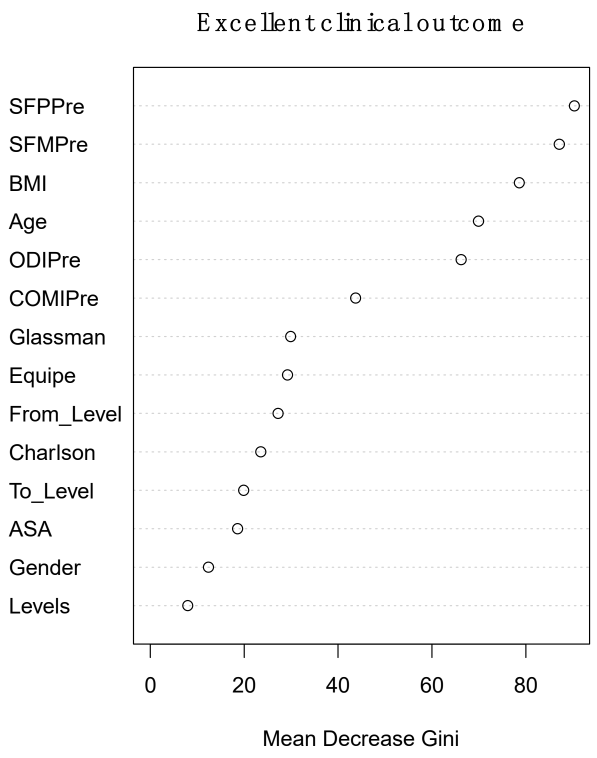

Figure S2. Mean Decrease Gini for Excellent Clinical Outcome. Glassman: Glassman classification data; Equipe: surgical team; BMI: body mass index; ODIPre: pre-operative; Oswestry disability index; COMIPre: pre-operative core outcome measures index; SFPPre: pre-operative physical component score of the short form-36; SFMPre: pre-operative men-tal component score of the short form-36; CCI: Charlson comorbidity index. From_level: Upper instrumented vertebra; To_level: lower level of instrumented vertebra. ASA: American Society of Anesthesiologists Score.
